# Acceptability and usability of Drugs4dent^®^, a dental medicines decision tool – a pilot study

**DOI:** 10.1186/s12903-025-06137-5

**Published:** 2025-05-22

**Authors:** Leanne Teoh, Ruby Biezen, Marietta Taylor, Richard O. Sinnott, Michael J. McCullough

**Affiliations:** 1 Melbourne Dental School, University of Melbourne, 720 Swanston Street, Carlton, VIC Australia; 2 Department of General Practice and Primary Care, University of Melbourne, Medical Building, Grattan Street, Melbourne, VIC Australia; 3 Melbourne eResearch Group, Faculty of Engineering and Information Technology, University of Melbourne, Level 5, Melbourne Connect, 700 Swanston Street, Melbourne, VIC Australia

**Keywords:** Digital health, Prescribing tool, Clinical decision, Dental prescribing, Dental decision tool

## Abstract

**Background:**

While drugs have a limited role in the management of dental presentations, Australian dentists have high rates of inappropriate prescribing of antibiotics. There is also a lack of relevant drug resources for dentists in Australia. Our team developed Drugs4dent^®^, a medicines decision support tool, that provides dentists with relevant drug knowledge, assists with appropriate prescribing and provides safety checks to reduce prescribing errors. The aim of this pilot study was to improve Drugs4dent^®^ with focus groups of dentists, and assess the acceptability, usability, and user experience, of Drugs4dent^®^.

**Methods:**

Focus groups of ten dentists were established to inform the improvement of Drugs4dent^®^. Acceptability and usability testing of Drugs4dent^®^ was then undertaken with a further ten dentists using interviews and a survey. The survey was based on the Framework for Acceptability and System Usability Scale. Inductive thematic analysis was undertaken using Nvivo for the focus groups and interviews, and descriptive statistics for analysis of survey results.

**Results:**

Dentists from the focus group and interviews found the content of Drugs4dent^®^ acceptable and useful for dentistry, recognising that similar drug information is currently not available. The majority agreed that Drugs4dent^®^ would improve their ability to prescribe according to guidance. Participants reported Drugs4dent^®^ was intuitive, and information was easy to locate. Most dentists preferred Drugs4dent^®^ integrated with their dental practice software. In the absence of this functionality, they preferred Drugs4dent^®^ as a standalone resource, without needing to input patient data. Drugs4dent^®^ was subsequently commercialised with MIMS Australia, to create the decision support tool: MIMS Drugs4dent^®^.

**Conclusions:**

Drugs4dent^®^ is the first dental medicines decision tool in Australia. The high acceptability and usability of the tool, and subsequent commercialisation indicates that MIMS Drugs4dent^®^ has substantial promise for the future, and can transform access to relevant drug information for Australian dentists.

**Supplementary Information:**

The online version contains supplementary material available at 10.1186/s12903-025-06137-5.

## Background

Dental pain and infection are common clinical presentations managed by dental practitioners, and generally require dental treatment, such as root canal treatment or tooth extraction, to address the cause of infection [1]. Medicines, such as antibiotics and pain relief, are only adjunctive to dental treatment and therefore have limited need in dentistry [1]. Despite this, high rates of inappropriate prescribing and prescribing errors have been demonstrated amongst dental practitioners, with antibiotics and opioids being most commonly prescribed [2–5].

Dental antibiotic prescribing accounts for 10% of all prescribed antibiotics worldwide, but up to 80% of dental antibiotics are prescribed inappropriately, for both therapeutic and prophylactic reasons [2, 5–7]. Inappropriate antibiotic prescribing contributes to the global public health problem of antimicrobial resistance, which is responsible for an estimated 700,000 deaths worldwide annually [8].

Opioids with/without paracetamol are the first choice for 27% of Australian dentists for managing dental pain for a surgical tooth extraction, despite opioids not being recommended as first line analgesia, as well as having limited effectiveness [2]. A longitudinal study has demonstrated that dental opioid prescribing has increased in recent years in Australia [4]. Opioid misuse is a major public health issue in Australia and the United States [4, 9, 10]. A cross-sectional study showed that 16% of dental opioid prescriptions in adults were linked to the development of persistent opioid use [9].

In addition to inappropriate prescribing of antibiotics and opioids, prescribing errors occur in dentistry, as demonstrated by an Australian case series analysis [11]. Furthermore, a qualitative study of dental prescribing practices revealed that dentists reported they lacked confidence in being familiar with the drugs in a patient’s medication history, and importantly in understanding how their patients’ medications could influence dental treatment to allow dental treatment to be conducted safely [12]. Dentists reported there were insufficient readily accessible drug resources specific for dentistry that describe the potential impact of medications on dental treatment [12, 13].

Decision support tools are increasingly implemented throughout healthcare to assist practitioners in improving prescription accuracy, optimising drug dosages and providing checks such as drug-drug or drug-allergy alerts. They can provide timely drug information, often designed to be accessed at point-of-care, and can be embedded into patient management software to enable individualised health care decisions. These systems have been used as interventions to improve medication safety and reduce prescribing errors [14, 15]. While these systems are extensively used in medicine and pharmacy, they are not as common in dentistry [16].

To address inappropriate dental prescribing, as well as the lack of access to relevant drug resources for Australian dentists, our team developed a medicines decision tool, Drugs4dent^®^. Drugs4dent^®^ was developed to provide dentists with relevant drug knowledge, calculate paediatric doses, provide safety checks and assist dentists to prescribe according to Australian national guidelines, Therapeutic Guidelines Oral and Dental and the Australian Medicines Handbook [1, 13]. Drugs4dent^®^ was trialled in a before-and-after pilot study, which established proof-of-concept for Drugs4dent^®^. The pilot study demonstrated a 41% reduction in the number of antibiotics prescribed, where Drugs4dent^®^ was trialled together with education as an intervention [1, 13]. The antibiotic prescription accuracy was also improved in this pilot study, as Drugs4dent^®^ provided antibiotic regimen recommendations according to Australian guidance [1, 17]. Dentists reported an increased confidence in understanding how their patient’s medications affected dental treatment [13]. While Drugs4dent^®^ was well received, dentists requested more information about the drugs they prescribed, such as drug interactions, and safety of drugs in pregnancy and lactation.

Given the positive feedback and success of the previous study that established proof-of-concept, we conducted another pilot study with dentists to assist with improving Drugs4dent^®^. The aim of this prospective pilot study was to improve Drug4dent^®^ using focus groups of dentists, then assess the acceptability, usability, and user experience, of Drugs4dent^®^.

## Methods

### Study design

There were two parts to this prospective pilot study. The first part consisted of two focus groups of ten dental practitioners to assess and inform the improvement of Drugs4dent^®^, and to obtain feedback around the acceptability and usability of the tool. The second part consisted of testing of the improved Drugs4dent^®^ with ten different dental practitioners for acceptability and usability with both qualitative interviews and a quantitative survey. This study was reported according to the Consolidated Criteria for Reporting Qualitative Research (COREQ) for interviews and focus groups [18]. The COREQ checklist is shown in Supplementary Table 1. Ethical approval was obtained from the University of Melbourne Human Research Ethics Committee (ID: 25510).

### Part 1: Focus groups to inform the improvement of Drugs4dent^®^

Ten dentists were invited to participate in a focus group conducted over the video conferencing platform, Zoom. The dentists were divided into two groups for practical reasons. The principal researcher (LT) who developed the Drugs4dent^®^ prototype [13], and a researcher with extensive focus group experience (RB), facilitated the discussion.

Participants were provided access to Drugs4dent^®^ one month prior to the focus group and were given instructions (user guide and video) on its use.

The focus groups explored attitudes and perceptions of Drugs4dent^®^ around acceptability, usability, content, and integration of Drugs4dent^®^ with the clinical workflow. Participants were given the opportunity to suggest improvements. The duration of the focus groups was approximately one hour. Focus group questions were developed based on the feedback interviews conducted in the Drugs4dent^®^ proof-of-concept pilot study [13]. These are shown in Supplementary Fig. 1.

Based on the feedback, Drugs4dent^®^ was revised and improved by the Melbourne eResearch Group at the University of Melbourne.

### Part 2: Acceptability and usability testing of Drugs4dent^®^

Following the improvement of Drugs4dent^®^, acceptability and usability testing occurred with ten different dentists. Each dentist was provided access to Drugs4dent^®^ for one month. Dentists subsequently underwent an individual semi-structured interview with the principal researcher (LT) to obtain detailed qualitative feedback about their attitudes and perceptions of the acceptability, usability, content, and user experience with Drugs4dent^®^. Interviews were between 11 and 15 min duration, and were conducted face-to-face, via telephone, or via Zoom. A survey was provided through an anonymised Qualtrics link, and questions were based on the Framework for Acceptability and System Usability Scale [19, 20]. The interview questions and survey are included in Supplementary Figs. 2 and 3 respectively.

### Participants and procedures

Dentists were recruited from around Victoria, Australia based on their prior involvement with the Drugs4dent^®^ proof-of-concept pilot study, or through snowball sampling techniques. A mix of sex, years of clinical experience, and workplace location including various socio-economic index for areas (SEIFAs) were considered to ensure a representation of dentists were achieved [21]. SEIFA was developed by the Australian Bureau of Statistics, and ranks areas in Australia according to relative socio-economic advantage (higher scores) and disadvantage (lower scores) [21].

Participants were invited to participate by email. Written consent was obtained before commencement of the focus group or interviews. Data were audio recorded and transcribed verbatim by a transcription company, Pacific Transcription.

## Outcomes

The primary outcome of this study was the acceptability and usability of the improved Drugs4dent^®^ based on the qualitative analysis of focus groups and interview responses, and quantitatively using the Framework for Acceptability and System Usability Scale. The secondary outcome was to qualitatively evaluate the dentists’ user experience with Drugs4dent^®^.

### Analysis

Descriptive statistics were used to analyse the survey results for acceptability and usability testing. Demographic and survey responses were stratified by sex, years of experience, and a Likert scale was used for the responses. The information was presented by category and summarised using proportions.

Transcripts of the focus groups and interviews were coded by LT and RB independently to identify pertinent concepts and ideas, according to the feasibility and acceptability of the tool. A coding scheme was derived by grouping emerging concepts during data collection. Using a thematic approach, codes were then merged and grouped to form themes and subthemes. The coding schemes from both researchers were compared and refined, all discrepancies were resolved through discussion and consensus. This process was repeated for all focus groups and interviews. Themes were predetermined and matched to the proof-of-concept pilot study and the Framework for Acceptability and System Usability Scale [13, 19, 20]. Data were managed using NVivo (QSR International, Australia).

### About Drugs4dent^®^

**Fig. 1:**
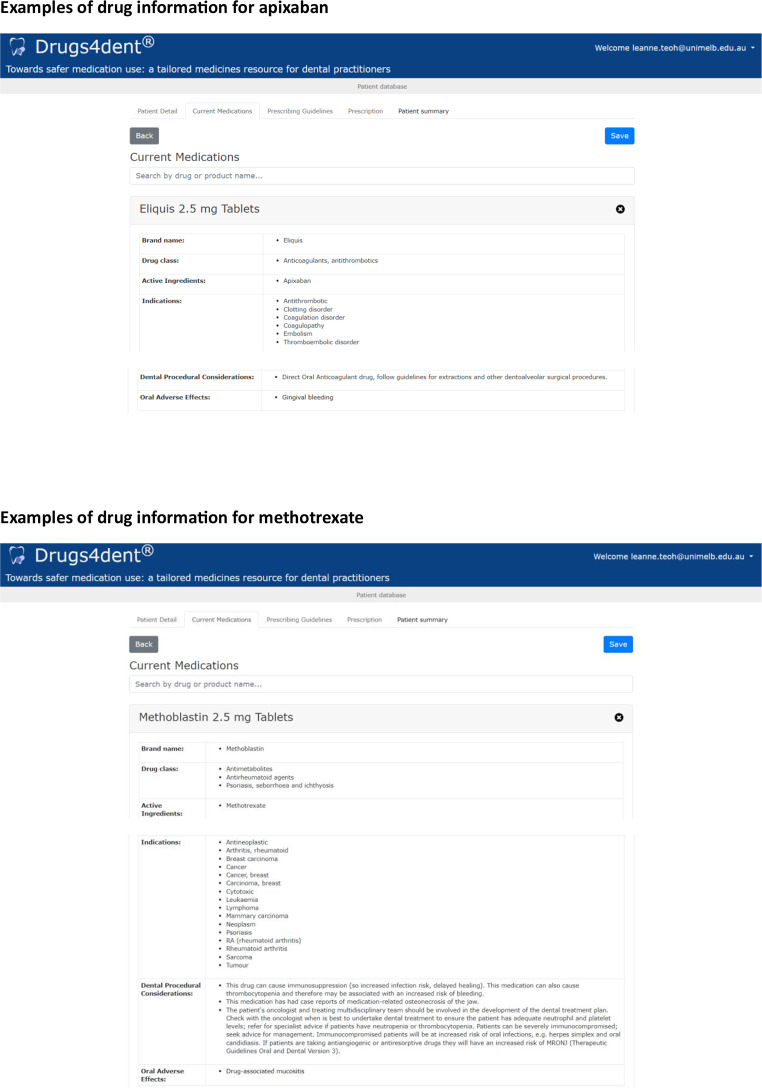
Examples of drug information presented in Drugs4dent^®^

Drugs4dent^®^ is a dental medicines decision tool, that provides tailored information about the effect of drugs on dental treatment, such as dental procedural considerations and oral adverse effects. Some examples of dental procedural considerations include drugs that contribute to bleeding risk and medication-associated osteonecrosis of the jaw. Examples of the drugs apixaban and methotrexate in the Drugs4dent^®^ user interface is shown in Fig. 1.

As drug information changes regularly, we collaborated with the drug compendia Monthly Index for Medical Specialities (MIMS) [22]. MIMS is a predominant provider of drug information in Australia, and content is from registered drug product information. Drugs4dent^®^ is currently integrated with MIMS using a targeted Representational State Transfer (ReST)-based application programming interface, to ensure drug information is current.

**Fig. 2:**
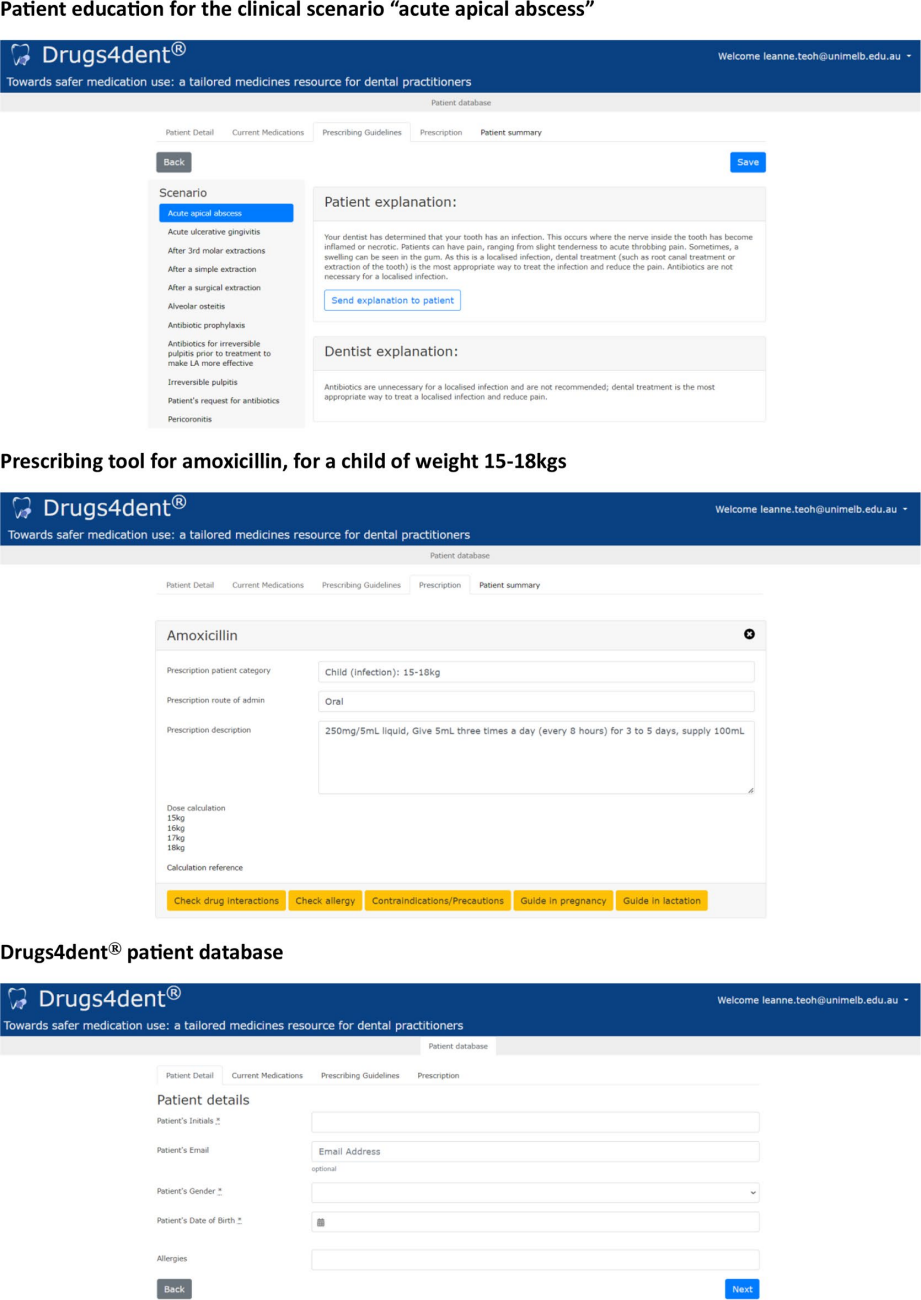
Examples of patient education, the prescribing tool and patient database in Drugs4dent^®^

Drugs4dent^®^ provides patient and dentist education about appropriate antibiotic use for various dental clinical scenarios. It also features a prescribing tool to assist dentists to prescribe in accordance with the Australian national guidelines, Therapeutic Guidelines Oral and Dental, and the Australian Medicines Handbook [1, 17]. This was achieved by providing recommendations according to guidance when dentists select the drug (e.g. antimicrobial, analgesic or anxiolytic) [1, 13]. Drugs4dent^®^ also has its own patient database, where individual patient details are recorded by the practitioner, and each patient has their own profile. Figure 2 shows examples of patient education, the prescribing tool, and the patient database in the Drugs4dent^®^ user interface.

### Role of the funding source

The funder of the study had no role in study design, data collection, data analysis, data interpretation or writing of the report.

## Results

Twenty-two dentists were approached for the focus groups and acceptability and usability testing, of which 20 agreed to participate (see Table 1 for participant demographics). The relatively even proportion of participants (male: *n* = 9), varied SEIFA of practice locations, and years of clinical experience reflects similar demographic indicators for dentists practising in Australia, as indicated by the Australian Health Practitioner Regulation Agency [23].

**Table 1:** Demographic information

Demographic details	Focus group	Interviews
	*n*	*n*
Numbers of participants	10	10
Sex		
Male	3	6
Female	7	4
Years of experience		
< 10 years	2	1
11–20 years	4	8
≥ 20 years	4	1
SEIFA* of practice location		
Low – 1, 2, 3	1	3
Middle – 4, 5, 6, 7	5	3
High – 8, 9, 10	4	4

*Socio-economic index for areas

Most suggestions for improvement during the focus groups were minor, relating to the layout and access to information. Common sub-themes emerged from the focus groups, interviews and acceptability and usability survey. These were incorporated into the overlying themes of acceptability, usability and user experience. Therefore, the results from Parts 1 and 2 are presented together.

### Acceptability

From the survey results, the majority of participants found Drugs4dent^®^ improved their skills and knowledge of appropriate prescribing (see Table 2). They also found the tool practical for dental clinical use.

**Table 2:** Survey responses

**Demographic Information**			
	**Female**	**Male**	
*n*=10 Sex	40%	60%	
	**<10 years**	**11-20 years**	**30+years**
Years of Experience	10%	80%	10%
**Acceptability of Drugs4dent** ^®^			
	**Uncomfortable**	**No opinion**	**Comfortable**
How comfortable did you feel with using Drugs4dent^®^?	10%	0%	90%
	**A lot** **of effort**	**No opinion**	**A little** **effort**
How much effort did it take to use Drugs4dent^®^?	20%	0%	80%
	**Disagree**	**No opinion**	**Agree**
Drugs4dent^®^ has improved my ability to access dentally-relevant drug knowledge to assist with improved patient management with respect to medication use.	0%	10%	90%
Drugs4dent^®^ has improved my ability to prescribe appropriately, regarding the correct dose, duration and frequency.	0%	10%	90%
Drugs4dent^®^ has improved my ability to prescribe medicines accurately and safely.	0%	10%	90%
	**Unconfident**	**No opinion**	**Confident**
How confident did you feel about using Drugs4dent^®^?	10%	10%	80%
**Usability of Drugs4dent** ^®^			
	**Disagree**	**No opinion**	**Agree**
It was easy to learn to use Drugs4dent^®^	0%	20%	80%
It was easy for me to find the information I needed in Drugs4dent^®^	10%	10%	80%
Overall, I am satisfied with Drugs4dent^®^	10%	0%	90%
**Format and User Experience**			
	**Disagree**	**No opinion**	**Agree**
I would prefer to have Drugs4dent^®^ integrated in my dental practice management software.	10%	0%	90%
I would like Drugs4dent^®^ to also be available as an app that I could download onto my smart phone.	0%	20%	80%

“The level of detail was good. I really appreciate the dental procedural considerations and the oral adverse effects.” (Focus Group).

“I think it was a good collection of information. I think in comparison to other drug compendia it was much more relevant to a dentist.” P7(Interview; 14 years’ experience, SEIFA: 10).

“…It all lined up with what I’ve read in therapeutic guidelines for the recommendations for practitioners. So, it’s very easy to have that as a handy resource just to pop in and get the information, rather than having to search it elsewhere….” P4(Interview; 15 years’ experience, SEIFA: 8).

Access to relevant drug information was cited to be a benefit of the tool and dentists reported that Drugs4dent^®^ can become a valuable resource for clinical practice. Dentists recognised that similar information is currently not available in Australia, therefore it can provide an independent and evidence-based source of support for decision making.

“I found it really useful in terms of the information in regards to the dental procedural considerations. I thought that really catered towards dental practitioners specifically…to have an independent peer reviewed body that you can give a print-out of information to a patient say, this is the reason why I’m not doing it…it’s because this is from an independent evidence-based resource.” P4(Interview; 15 years’ experience, SEIFA: 8).

“…having pharmaceutical information with a dental focus is something that’s really lacking. I don’t think there is any other product that replicates that.” P8(Interview; 16 years’ experience, SEIFA: 6).

Majority of participants in the survey (90%) expressed that they were comfortable using Drugs4dent^®^ and found that little effort was needed for interaction and engagement (see Table 2). Participants agreed that Drugs4dent^®^ improved their ability to access relevant drug knowledge and would be helpful with clinical management of their patients. Most participants indicated that the tool would improve their ability to prescribe according to current guidelines, but also being able to check drug interactions and the safety of drugs in specific cohorts, such as patients who are pregnant or breastfeeding.

“That was really helpful there (drug interactions and allergy alerts), because especially, I used to have to just Google those things up especially if they had a lot of medications. Now, you can just click on that and it’ll show you, these will interact with these.” P1(Interview; 7 years’ experience, SEIFA: 2).

### Usability

Majority of participants agreed that Drugs4dent^®^ was intuitive to use and they were able to locate desired information easily, without having to reference the user guide (see Table 2).

“Yeah, I - well, I kind of looked at neither first (user guide or video) and found it was quite intuitive, so I didn’t really see the need for them.” (Focus group).

“Not much effort at all (effort to use Drugs4dent^®^ during clinical time). I mean, it’s just on the computer and I can just key in everything right there and then…there’s a very low learning curve involved so it was an easy-to-use program.” P3(Interview; 23 years’ experience, SEIFA: 10).

Overall, participants found Drugs4dent^®^ a valuable resource that would be used regularly during clinical practice, especially for unfamiliar medications or for managing patients with complex medical conditions.

“Potentially, it could become a really valuable resource. I would use it – saying daily might be overstating it, but I can see myself using it really regularly to look up…medications that I haven’t come across before.” P3(Interview; 23 years’ experience, SEIFA: 10).

“I’d probably say most of my patients have some kind of medical - underlying medical issues, so I’d probably (use Drugs4dent^®^) say about 40 or 50% of the time.” P6(Interview; 15 years’ experience, SEIFA: 10).

### Format and user experience

Some participants found accessing Drugs4dent^®^ as a standalone tool acceptable, as current drug resources are accessed in a similar way.

“I thought it was pretty easy to use, so it’s not difficult - I was happy with it being a standalone program.” (Focus group).

However, as Drugs4dent^®^ is designed to have its own patient database, most dentists felt that entering in patient details into a separate database would be an administrative burden. Consequently, most participants preferred Drugs4dent^®^ to be a standalone tool without a patient database.

*“The least user-friendly part of the website is the fact that it wasn’t integrated and that we needed to put in patient data that was already input into somewhere else.” P9(Interview; 15 years’ experience*,* SEIFA: 1)*.

*“I think the clinical workflow is fine…I would appreciate that other people would find it difficult*,* or tedious*,* to put in all the medications all of the time and having to create that patient database.” P7(Interview; 14 years’ experience*,* SEIFA: 10)*.

However, some participants preferred Drugs4dent^®^ to be fully integrated into their dental practice management software.

“If it’s integrated it just goes straight across, it’s quite easy and straightforward.” (Focus group).

“It probably would be more convenient if it was integrated into (dental practice software).” P5(Interview; 16 years’ experience, SEIFA: 7).

In terms of using Drugs4dent^®^ as a decision support tool, the majority of participants thought having an application would be helpful.

“I would definitely be wanting to use it as an app. I mean, often times you do have to field a phone call from a patient and organise something when you’re not in front of your computer. Being able to pull it up on the phone would be brilliant.” P4(Interview; 15 years’ experience, SEIFA: 8).

### Commercialisation outcome

With the high rate of acceptability and usability from participating dentists, successful negotiations occurred to commercialise Drugs4dent^®^ as a sub-licence to MIMS Australia. The outcome is to create a standalone dental medicines decision tool: MIMS Drugs4dent^®^. Given the challenges raised by dentists in this study with regards to having a separate patient database, it was decided to remove the patient database but retain the same format and content of dental-specific drug information, the prescribing tool and the associated safety checks within the MIMS Drugs4dent^®^ product.

## Discussion

Drugs4dent^®^ started as a concept to provide timely and accessible drug knowledge for dentists and to assist with prescribing. This study describes the progression to commercialisation of this landmark medicines decision tool for the Australian dental industry. Drugs4dent^®^ has been developed to address several unmet needs in dentistry, including timely access to relevant drug knowledge, and information pertaining to the impact of drugs on dental treatment. Calculation of drug doses for paediatric prescribing, safety features including drug and allergy interactions, and appropriateness of drugs in pregnancy and lactation are included, enabling improved prescribing. The high acceptability and usability of the tool by dentists in the present pilot study, suggests that Drugs4dent^®^ has substantial promise for uptake and can transform the access and availability of medicines decision support for Australian dentistry.

Adoption of decision support in dentistry is minimal, despite decision support being common in medicine, particularly in secondary and tertiary care. Various reasons exist, one of which is that most dental patient management software offers minimal diagnostic or decision support [16]. While some tools have been developed for dentistry to assist with diagnosis and management of caries, and paediatric dental trauma, uptake has been slow [16, 24]. Given the growing ageing population with patients retaining teeth for longer and increasing polypharmacy, dentists are faced with navigating larger amounts of patient, clinical and drug information, and patients with more complex medication histories [25]. Since drug information changes regularly, decision support at point-of-care helps clinicians to stay up-to-date, and provides targeted advice [25]. Current drug compendia in Australia provide non-specific, detailed drug information, most of which is not relevant for dentistry. Furthermore, oral adverse reactions to medications are difficult to locate in current drug compendia, as they do not have their own dedicated section in the product information, so tend to be scattered in different sections. For example, bruxism can be found in the psychiatric section, xerostomia in the gastrointestinal section, and taste disturbance in the special senses section. [26]. However, this is not consistent and can differ, and thus oral adverse effects can be difficult to locate [26]. Current drug compendia also does not capture the effects of drugs on dental procedures. MIMS Drugs4dent^®^ provides the Australian dental industry with an opportunity to access, for the first time, tailored dental drug information in a decision support tool.

Drugs4dent^®^ is designed to assist dental practitioners to prescribe according to Australian guidelines, improve prescription accuracy, calculate paediatric doses and provide safety alerts to decrease errors. While these features are already available in other drug compendia, the other novel facets of Drugs4dent^®^, including the prescribing tool with recommendations concordant with Australian dental guidelines, patient education about appropriate antibiotic use in the dental context, and dental-specific drug information including dental procedural considerations and oral adverse effects, are not readily available elsewhere. Drugs4dent^®^ thus provides all these features in one tool, and these were positively accepted by dentists as outlined in this study. Australian case reports of dental medication errors have been published, highlighting errors due to inappropriate drug selection, drug-drug or drug-allergy interactions [11]. Having timely, accessible and relevant drug and prescribing information has been shown to reduce medication and prescribing errors, optimise prescribing regimens and drug selection, reduce adverse effects and thus improve patient safety [27, 28]. Medication safety is a clear benefit of Drugs4dent^®^, noting that utilisation and uptake are crucial components to realise these benefits [28].

While MIMS Drugs4dent^®^ is developed as a standalone tool, optimal uptake and improved appropriateness of prescribing will likely be increased with integration into practice management software. A systematic review of prescribing decision support integrated with patient electronic records demonstrated that of the 41 randomised controlled trials, 25 trials reported success at changing practitioner behaviour and five showed improvements in patient outcomes [14]. Features of medicines decision support considered critical for improving clinical practice include automation of support integrated with the clinician workflow, and that advice is provided at the time and location of decision making [29]. Integration of MIMS Drugs4dent^®^ with dental practice software would enable timely and individualised patient advice at point-of-care, and would be the next step to further enhance the functionality and uptake of MIMS Drugs4dent^®^.

Provider attitudes and behaviour influence the acceptability and uptake of decision support tools. The positive attitudes towards utilisation of Drugs4dent^®^ in clinical practice by dentists in the present study and the intuitive nature of the tool were promising with regards to future potential uptake. A survey of provider attitudes showed factors such as satisfaction with the electronic health record, strong evidence underpinning the tool, functional reliability, and a dentists’ personal technology adoption mindset, all influenced increased satisfaction and positive implementation of decision support [16]. While the current study did not fully investigate barriers and enablers of uptake, these attitudes are important to consider when deploying Drugs4dent^®^ in clinical practice. Further investigations can be targeted to understanding provider attitudes in Australian dental practice to influence optimal uptake of Drugs4dent^®^.

In the context of inappropriate dental prescribing, decision tools in primary care have been shown to be effective as antimicrobial stewardship interventions [30]. The previous pilot study that demonstrated proof-of-concept also showed Drugs4dent^®^ and education to be an effective antibiotic stewardship intervention [13]. In primary care medicine, decision support systems have been associated with significant improvements in appropriate antibiotic prescribing in a systematic review, although automated systems that provided information at the time required in the clinical workflow appeared to be more effective [30]. In dentistry, a decision tool providing personalised pain management recommendations to reduce inappropriate dental opioid prescribing showed that providers who accessed the application found it beneficial and time saving [31]. Future research should involve auditing of prescribing with MIMS Drugs4dent^®^ and assessment of appropriateness according to national guidelines.

This study has some limitations. As the focus groups and interviews were conducted with the principal researcher (LT), dentists may have provided favourable responses. To address this, the acceptability and usability survey was anonymous to allow dentists the opportunity to provide unbiased responses and also to triangulate the results. The selection bias and small number of participants was also a limitation, as dentists who participated were likely interested in drug information and so may see the benefit in tools that assist with medication and prescribing safety. One potential obvious limitation is that two authors are co-inventors of MIMS Drugs4dent^®^. Although there are questions regarding reflexivity due to this background in the study, every attempt was made to analyse the data in this pilot study using robust and unbiased methodology. The other authors had no involvement in the development of this tool which was anticipated would mitigate any potential bias.

Given the positive findings from this pilot study for improving Drugs4dent^®^, further research for MIMS Drugs4dent^®^ includes wider dissemination of the tool, and a large-scale implementation and evaluation study for dental practitioners in Australia and New Zealand. As dentists reported preferencing integration of Drugs4dent^®^ with practice management software, future plans involve investigating this possibility to enable the provision of patient-specific and timely presentation of drug information at point-of-use. Inclusion of electronic prescribing software is also a consideration for future incorporation into the tool to further enhance its usability.

## Conclusions

This study describes the improvement of Drugs4dent^®^, and its progression from inception through to commercialisation. The high acceptability and usability of the tool, and subsequent commercialisation indicates that MIMS Drugs4dent^®^ has substantial promise for use in clinical practice. With features that enable timely access to dental-specific drug information, patient education about appropriate antibiotic use, safety alerts, and assisting with prescribing according to guidelines, MIMS Drugs4dent^®^ marks the beginning of prescribing and medicines decision support for dentistry in Australia.

## Electronic supplementary material

Below is the link to the electronic supplementary material.

Supplementary Material 1

## Data Availability

After publication, de-identified data will be made available upon reasonable request to the corresponding author.

## Declarations

### Competing of interests

LT and MMc are both recognised as inventors of Drugs4dent^®^ at the University of Melbourne. MIMS Drugs4dent^®^ is a commercial product, from which LT and MMc will receive royalties.
