# Supplementary material for: Acceptability and usability of Drugs4dent®, a dental medicines decision tool – a pilot study
Source: BMC Oral Health. 2025 May 22;25:766. doi: 10.1186/s12903-025-06137-5 (PMC12096631; doi:10.1186/s12903-025-06137-5)
Supplement: Supplementary file 1 — Supplementary Material 1 [file 12903_2025_6137_MOESM1_ESM.docx]

**Supplementary Table 1: Consolidated criteria for reporting qualitative research (COREQ) checklist for interviews and focus groups**

| **Item** | **Guide questions/description** | **Page** |
| --- | --- | --- |
| ***Domain 1: Research team and reflexivity*** | | |
| *Personal Characteristics* | | |
| 1. Interviewer/facilitator | Which author/s conducted the interview or focus group? | Methods,  Page 6 |
| 2. Credentials | What were the researcher’s credentials? E.g. PhD, MD | Title page; Methods,  Page 6 |
| 3. Occupation | What was their occupation at the time of the study? | N/A |
| 4. Gender | Was the researcher male or female? | N/A |
| 5. Experience and training | What experience or training did the researcher have? | Methods  Page 6 |
| *Relationship with participants* | | |
| 6. Relationship established | Was a relationship established prior to study commencement? | Methods  Page 6 |
| 7. Participant knowledge of the interviewer | What did the participants know about the researcher? e.g. personal goals, reasons for doing the research | Discussion, Page 14 |
| 8. Interviewer characteristics | What characteristics were reported about the interviewer/facilitator? e.g. Bias, assumptions, reasons and interests in the research topic | Discussion, Page 14 |
| ***Domain 2: study design*** | | |
| *Theoretical framework* | | |
| 9. Methodological orientation and Theory | What methodological orientation was stated to underpin the study? e.g. grounded theory, discourse analysis, ethnography, phenomenology, content analysis | Methods,  Page 7 |
| *Participant selection* |  |  |
| 10. Sampling | How were participants selected? e.g. purposive, convenience, consecutive, snowball | Methods,  Page 6 |
| 11. Method of approach | How were participants approached? e.g. face-to-face, telephone, mail, email | Methods,  Page 7 |
| 12. Sample size | How many participants were in the study? | Results,  Page 8 |
| 13. Non-participation | How many people refused to participate or dropped out? Reasons? | Results,  Page 8 |
| *Setting* | | |
| 14. Setting of data collection | Where was the data collected? e.g. home, clinic, workplace | Methods,  Page 8 |
| 15. Presence of non-participants | Was anyone else present besides the participants and researchers? | Methods  Page 6 |
| 16. Description of sample | What are the important characteristics of the sample? e.g. demographic data, date | Results  Page 8,  Table 1 |
| *Data collection* | | |
| 17. Interview guide | Were questions, prompts, guides provided by the authors? Was it pilot tested? | Methods Page 6, Supplementary Figure 1 |
| 18. Repeat interviews | Were repeat interviews carried out? If yes, how many? | N/A |
| 19. Audio/visual recording | Did the research use audio or visual recording to collect the data? | Methods,  Page 6 |
| 20. Field notes | Were field notes made during and/or after the interview or focus group? | Methods,  Page 7 |
| 21. Duration | What was the duration of the interviews or focus group? | Methods Page 6 |
| 22. Data saturation | Was data saturation discussed? | N/A |
| 23. Transcripts returned | Were transcripts returned to participants for comment and/or correction? | N/A |
| ***Domain 3: analysis and findings*** | | |
| *Data analysis* | | |
| 24. Number of data coders | How many data coders coded the data? | Methods,  Page 7 |
| 25. Description of the coding tree | Did authors provide a description of the coding tree? | N/A |
| 26. Derivation of themes | Were themes identified in advance or derived from the data? | Methods,  Page 7 |
| 27. Software | What software, if applicable, was used to manage the data? | Methods,  Page 7 |
| 28. Participant checking | Did participants provide feedback on the findings? | Results,  Pages 9-11 |
| *Reporting* | | |
| 29. Quotations presented | Were participant quotations presented to illustrate the themes / findings? Was each quotation identified? e.g. participant number | Results,  Pages 9-11 |
| 30. Data and findings consistent | Was there consistency between the data presented and the findings? | Results,  Pages 9-11 |
| 31. Clarity of major themes | Were major themes clearly presented in the findings? | Results,  Pages 9-11 |
| 32. Clarity of minor themes | Is there a description of diverse cases or discussion of minor themes? | Results  Pages 9-11 |

**Supplementary Figure 1: Focus group discussion questions**

**Focus groups to inform the upgrade of Drugs4dent^®^ V3.1**

Thank you for participating in this study trialling Drugs4dent^®^ linked to EXACT. We appreciate your feedback to further improve Drugs4dent^®^.

USER GUIDE AND VIDEO

- Which one was easier to use?
- Was there too much/too little information?
- Would you prefer having both, or just one?
- What can be improved?

CONTENT OF DRUGS4DENT^®^

**Current Medications**

For the section titled ‘Current Medications’, is there any other information you would like to know about drugs a patient is taking?

- Was the information clinically relevant?
- Would you like to see more details about medications, e.g. use of anti-thrombotics? Osteoporosis? MRONJ?
- Was this easy to understand/navigate?

**Prescribing guidelines**

The section on Prescribing Guidelines was aimed to help assist dentists with patient expectations.

- If you did not use this section, why not?
- Would you email any of the information to your patients?
- Are the patient explanations clear?
- Would you prefer prescribing competencies included in this section?

**Prescription**

The section on Prescription was aimed to help dentists prescribe according to the dental Therapeutic Guidelines, as well as assist with prescribing for children.

- Did you find this information helpful? If so, why? If not, why not?
- Would you like to be able to print your prescription from this section?
- Did you find the drug interactions easy to use and interpret?
- Did you find the allergy interactions helpful?

**General comments about content of Drugs4dent**

Is there any other information you think should be included in Drugs4dent^®^?

- Is there any other feedback you can give us about Drugs4dent^®^?
- Would you like to have included prescribing law in dentistry here as well?

CLINICAL WORKFLOW

Which sections do you use the most?

How did you find the clinical workflow and the order of information presented?

- Do you prefer to navigate through the tabs at the top, or next/back buttons?
- Did you find having to save to the end a hindrance to the workflow?
- How do you find the PDF at the end?

How easy/hard was it to search for patients?

Do you want to be able to print your prescriptions?

**Integration**

We are planning to eventually integrate Drugs4dent^®^ into dental software. Integration will involve moving patient details across, and enabling electronic prescriptions to be written and printed through Drugs4dent^®^. Your prescriptions and details about medications will also be stored under the individual patient file in EXACT. Would this be helpful, or would you prefer to use Drugs4dent^®^ as a standalone system?

**Supplementary Figure 2: Interview questions**

**Interview schedule: Validation testing of Drugs4dent^®^ V3.2**

Thank you for participating in this study trialling Drugs4dent^®^ linked to EXACT. We appreciate your feedback to further improve Drugs4dent^®^.

ABOUT YOU

Q1. What is your gender?

- Male
- Female
- Prefer not to say

Q2. How many years of clinical experience do you have?

- <10 years
- 11-20 years
- 21-30 years
- >30 years

Q3. What is the postcode of your work location where you used Drugs4dent^®^?

(Written response)

ABOUT DRUGS4DENT

Q4. How often did you use Drugs4dent^®^?

- Once <5 patients
- Once every 5-10 patients
- Once every 10-20 patients
- Once >20 patients

Q5. How did you find the clinical workflow and the order of information presented?

- Please let us know if there can be improvements
- Did you find having to save to the end a hindrance to the workflow?

Q6. For the section titled ‘Current Medications’, is there any other information you would like to know about drugs a patient is taking?

- Was the information clinically relevant?
- Would you like to see more details about medications, e.g. use of anti-thrombotics? Osteoporosis? MRONJ?

Q7. The section on Prescribing Guidelines was aimed to help assist dentists with patient expectations. Did you find you needed to use this section?

- If you did not use this section, why not?
- Would you email any of the information to your patients?
- Are the patient explanations clear?

Q8. The section on Prescription was aimed to help dentists prescribe according to the dental Therapeutic Guidelines, as well as assist with prescribing for children.

- Did you find this information helpful? If so, why? If not, why not?
- Would you like to be able to print your prescription from this section?
- Did you find the drug interactions easy to use and interpret?
- Did you find the allergy interactions helpful?

Q9. We are planning to eventually integrate Drugs4dent^®^ into dental software. Integration will involve moving patient details across, and enabling electronic prescriptions to be written and printed through Drugs4dent^®^. Your prescriptions and details about medications will also be stored under the individual patient file in EXACT. Would this be helpful, or would you prefer to use Drugs4dent^®^ as a standalone system?

- Yes, I think integration would be better
- No, I prefer Drugs4dent^®^ as a standalone system

Q10. Is there any other information you think should be included in Drugs4dent^®^?

Q11. Is there any other feedback you can give us about Drugs4dent^®^?

**Supplementary Figure 3: Survey questions and responses**

|  | **DEMOGRAPHIC INFORMATION** |  |  |  |  |  |
| --- | --- | --- | --- | --- | --- | --- |
|  | *n=10* | **Female** | **Male** |  |  |  |
| Q1 | Sex | 30% | 70% |  |  |  |
|  |  | **<10 Years** | **11 to 20** | **30+years** |  |  |
| Q2 | Years of Experience | 10% | 80% | 10% |  |  |
| Q3 | Location | Major City |  |  |  |  |
|  | **PART 1: ACCEPTABILITY OF DRUGS4DENT®** |  |  |  |  |  |
|  |  | **Very Uncomfortable** | **Uncomfortable** | **No Opinion** | **Comfortable** | **Very Comfortable** |
| Q4 | How comfortable did you feel with using Drugs4dent®? | 0% | 10% | 0% | 70% | 20% |
| Q5 | How comfortable did you feel receiving the education program? | 0% | 0% | 0% | 50% | 50% |
|  |  | **Huge Effort** | **A lot of effort** | **No Opinion** | **A little effort** | **No effort at all** |
| Q6 | How much effort did it take to use Drugs4dent®? | 0% | 20% | 0% | 80% | 0% |
| Q7 | How much effort to engage with D4D education program? | 0% | 0% | 0% | 70% | 30% |
|  |  | **Strongly disagree** | **Disagree** | **No opinion** | **Agree** | **Strongly agree** |
| Q8 | Drugs4dent® has improved my ability to access dentally-relevant drug knowledge (e.g. which drugs cause MRONJ, increased bleeding risk etc) to assist with improved patient management with respect to medication use. | 0% | 0% | 10% | 70% | 20% |
|  | Drugs4dent® has improved my ability to prescribe appropriately, regarding the correct regimen (dose, duration and frequency) according to guidelines. | 0% | 0% | 10% | 60% | 30% |
|  | The education provided has improved my ability to prescribe appropriately, regarding the correct regimen (dose, duration and frequency) according to guidelines. | 0% | 0% | 10% | 50% | 40% |
|  | Drugs4dent® has improved my ability to prescribe medicines accurately and safely, with respect to drug and allergy interactions, pregnancy and breastfeeding considerations. | 0% | 0% | 10% | 60% | 30% |
|  | It is clear to me how Drugs4dent® will help me access dentally-relevant drug knowledge and prescribe more safely. | 0% | 0% | 0% | 60% | 40% |
|  | Engaging with Drugs4dent® chairside interfered with my priorities. | 0% | 60% | 20% | 20% | 0% |
|  |  | **Very unconfident** | **Unconfident** | **No opinion** | **Confident** | **Very confident** |
| Q9 | How confident did you feel about using Drugs4dent®? | 0% | 10% | 10% | 70% | 10% |
|  |  | **Completely unacceptable** | **Unacceptable** | **No opinion** | **Acceptable** | **Completely acceptable** |
| Q10 | How acceptable was the intervention of Drugs4dent® and education to you? | 0% | 0% | 10% | 70% | 20% |
|  | **PART 2: USABILITY OF DRUGS4DENT®** |  |  |  |  |  |
|  |  | **Strongly disagree** | **Disagree** | **No opinion** | **Agree** | **Strongly agree** |
| Q11 | Overall, I am satisfied with how easy it is to use Drugs4dent® | 0% | 30% | 0% | 70% | 0% |
|  | I was able to access information I need quickly using Drugs4dent® | 0% | 30% | 30% | 40% | 0% |
|  | It was easy to learn to use Drugs4dent® | 0% | 0% | 20% | 70% | 10% |
|  | Whenever I made a mistake using Drugs4dent®, I could recover easily and quickly. | 0% | 10% | 10% | 70% | 10% |
|  | It was easy for me to find the information I needed in Drugs4dent® | 0% | 10% | 10% | 70% | 10% |
|  | The organization of information on Drugs4dent® was clear. | 0% | 0% | 10% | 90% | 0% |
|  | The interface (e.g. screens, graphics, language) of Drugs4dent® was pleasant and I liked it. | 0% | 20% | 40% | 40% | 0% |
|  | Drugs4dent® has all the functions and capabilities I need for accessing drug knowledge and appropriate prescribing. | 0% | 10% | 30% | 60% | 0% |
|  | Overall, I am satisfied with Drugs4dent® | 0% | 10% | 0% | 90% | 0% |
|  | **PART 3. FORMAT AND USER EXPERIENCE** |  |  |  |  |  |
|  |  | **Strongly disagree** | **Disagree** | **No opinion** | **Agree** | **Strongly agree** |
| Q12 | If Drugs4dent® was to remain in its current user format, I would prefer to have it integrated in my dental practice management software (Drugs4dent®’s patient database is linked directly to the specific patient in my dental practice software). | 0% | 10% | 0% | 70% | 20% |
|  | If Drugs4dent® cannot be integrated to my dental practice software, I would prefer Drugs4dent® NOT to be linked to a specific patient (such that there is no patient database), while still providing the same content (drug information, patient education and guide to prescribing, drug interactions, allergy interactions and pregnancy/breastfeeding information). | 0% | 0% | 0% | 50% | 50% |
|  | If Drugs4dent® did not have its own patient database (NOT linked to a specific patient), I would like it to also be available as an app that I could download onto my smart phone. | 0% | 0% | 20% | 60% | 20% |
|  | If Drugs4dent® cannot be integrated to my dental practice management software, I would still be happy to use it in its current format. | 0% | 20% | 10% | 70% | 0% |
| Q13 | If Drugs4dent® cannot be integrated to my dental practice software, I would prefer Drugs4dent® NOT to be linked to a specific patient (such that there is no patient database), while still providing the same content (drug information, patient education and guide to prescribing, drug interactions, allergy interactions and pregnancy/breastfeeding information). | 0% | 0% | 0% | 50% | 50% |
| Q14 | If Drugs4dent® did not have its own patient database (NOT linked to a specific patient), I would like it to also be available as an app that I could download onto my smart phone. | 0% | 0% | 20% | 60% | 20% |
| Q15 | If Drugs4dent® cannot be integrated to my dental practice management software, I would still be happy to use it in its current format. | 0% | 20% | 10% | 70% | 0% |
